# A Curcumin-Loaded Liposomal Photodynamic Nanosystem for Effective Treatment of Bacterial Wound Infections

**DOI:** 10.3390/pharmaceutics18091122

**Published:** 2026-09-07

**Authors:** Huiya Chen, Xiaoyu Zhao, Minyan Zhang, Yu Zhang, Yuhan Huang, Hanqi Zhang, Min Lin, Lechun Lyu, Dan Xu

**Affiliations:** 1 Department of Dermatology, First Affiliated Hospital of Kunming Medical University, Kunming 650032, China; chenhuiya23@163.com (H.C.); zxyu124@163.com (X.Z.); 20230472@kmmu.edu.cn (M.Z.); 17316787516@163.com (Y.H.); 20240511@kmmu.edu.cn (H.Z.); 2 School of Rehabilitation and Yunnan Key Laboratory of Plateau Thermal Medical Rehabilitation and Wellness, Kunming Medical University, Kunming 650500, China; zyxwz2803@163.com; 3 State Key Laboratory of Complex Nonferrous Metal Resources Clean Utilization, Faculty of Metallurgical and Energy Engineering, Kunming University of Science and Technology, Kunming 650093, China; linmin@kust.edu.cn

**Keywords:** infectious wounds, curcumin-loaded liposomes, photodynamic therapy

## Abstract

**Background/Objectives:** Bacterial wound infections remain a significant clinical burden because of prolonged healing and the increasing prevalence of multidrug-resistant bacteria. This study aimed to develop a curcumin-loaded liposomal nanosystem (Lipo-Cur) for light-triggered photodynamic antibacterial therapy and enhanced wound repair. **Methods:** Lipo -Cur was prepared and characterized in terms of morphology, particle size, dispersibility, drug en-capsulation, curcumin solubility, and photostability. Reactive oxygen species generation under 430 nm blue-light irradiation was evaluated. The antibacterial activity of Lipo-Cur against Staphylococcus aureus and Escherichia coli was assessed in vitro. Its effects on the proliferation, migration, and colony formation of L929 and HaCaT cells were also examined. Therapeutic efficacy and biosafety were further evaluated in a murine S. aureus-infected wound model. **Results:** Lipo-Cur exhibited a near-spherical morphology with an average diameter of 134.8 nm, excellent dispersibility, high drug encapsulation, and improved curcumin solubility and photostability. Under 430 nm blue-light irradiation, Lipo-Cur efficiently generated reactive oxygen species. It exhibited minimum inhibitory concentrations of 100 and 125 μg/mL against *S. aureus* and *E. coli*, respectively, and achieved bactericidal rates exceeding 95%, outperforming free curcumin. Lipo-Cur also significantly enhanced L929 and HaCaT cell proliferation, migration, and colony formation, indicating favorable biocompatibility. In the murine infect-ed-wound model, Lipo-Cur combined with blue-light irradiation (Lipo-Cur-BL^+^) achieved 93.76 ± 1.56% wound closure by day 12, accelerated tissue regeneration, reduced inflammation, and caused no evident systemic toxicity or hemolysis. **Conclusions:** These findings demonstrate that Lipo-Cur is a safe and effective photodynamic antibacterial platform with considerable potential for treating bacterially infected wounds and promoting wound healing.

## 1. Introduction

Chronic infectious wounds, particularly those caused by multidrug-resistant (MDR) bacteria-such as diabetic foot ulcers, burn wounds, and postoperative infections-pose a growing global public health challenge [1,2]. These wounds are difficult to treat due to prolonged healing times, high recurrence rates, and frequent biofilm formation, which significantly reduces the efficacy of conventional therapies. Staphylococcus aureus (*S. aureus*) and its methicillin-resistant variant (MRSA) are the most prevalent pathogens, responsible for over 80% of skin and soft tissue infections. Alarmingly, more than 30% of patients experience recurrence within three months post-treatment [3]. Biofilms act as protective barriers against both antibiotics and immune responses, increasing bacterial resistance by up to 1000-fold [4]. Antibiotic-resistant infections already result in hundreds of thousands of deaths annually, and this number is projected to reach 10 million by 2050, with estimated global economic losses exceeding USD 100 trillion [5]. Current clinical strategies primarily involve systemic antibiotics, debridement, and wound dressings. However, inappropriate antibiotic use accelerates the emergence of drug-resistant strains, complicating treatment and increasing healthcare burdens [6]. Biofilm barriers further hinder drug penetration, diminishing therapeutic outcomes. Systemic therapies often cause adverse effects such as hepatotoxicity, nephrotoxicity, and microbiota imbalance. Conventional dressings offer only passive protection and generally lack antibacterial or regenerative functions [7]. The combined impact of MDR pathogens, biofilm-mediated resistance, and localized hypoxia represents a critical limitation of traditional treatments. There is an urgent need for innovative therapeutic approaches that offer localized delivery, potent antimicrobial activity, promotion of tissue regeneration, and a low risk of resistance development.

Photodynamic therapy (PDT) has gained increasing attention as an effective antimicrobial approach for infectious wound treatment, due to its high bactericidal efficiency, low risk of resistance, controllable selectivity, and compatibility with nanocarrier systems. It has been successfully applied against drug-resistant pathogens, including MRSA, *Escherichia coli* (*E. coli*), and Pseudomonas aeruginosa, with notable efficacy in disrupting established biofilms [8,9,10]. PDT operates through the light-induced activation of a photosensitizer, producing reactive oxygen species (ROS) such as singlet oxygen and hydroxyl radicals. These ROS cause irreversible damage to bacterial membranes, DNA, and proteins via mechanisms that bypass traditional antibiotic pathways, thus minimizing the likelihood of resistance development [4]. The antibacterial effect of PDT can be precisely modulated by controlling light wavelength, irradiation time, and photosensitizer concentration, enabling spatiotemporal targeting and reducing collateral damage to surrounding healthy tissues [1]. In addition to its antimicrobial function, PDT facilitates wound healing by promoting fibroblast proliferation, angiogenesis, and collagen synthesis. It also enhances innate immunity by stimulating neutrophils to release neutrophil extracellular traps (NETs), further contributing to local infection control [4,11].

Photosensitizers are essential to the therapeutic efficacy of PDT. Stable and targeted photosensitizers can significantly enhance the efficacy of PDT. The fundamental components of PDT include the photosensitizer, a light source with a wavelength matching the absorption spectrum of the photosensitizer, and sufficient oxygen availability [12]. Currently, photosensitizers have undergone three generations of iterative development. However, third-generation photosensitizers have seen limited clinical application due to the complexity and specificity of their functional modifications and conjugates [13]. Therefore, it is particularly urgent to design and develop new photosensitizers for photodynamic treatment of infectious wounds.

Curcumin (Cur), a polyphenolic compound derived from the rhizome of Curcuma longa, possesses diverse biological properties, including antibacterial, antioxidant, anti-inflammatory, and photosensitizing activities, making it an attractive natural photosensitizer. Increasing evidence indicates that Cur-based PDT provides notable antibacterial efficacy [14], with marked inhibitory effects against both Gram-positive bacteria (e.g., *S. aureus*) and Gram-negative bacteria (e.g., *E. coli*) [15]. Its stability and controlled release under acidic or infectious microenvironments can further enhance its bioadaptability and targeting capacity [2]. Cur exerts antibacterial effects through multiple mechanisms. It disrupts bacterial membrane integrity, suppresses cell division, induces protein and DNA damage [16,17], and inhibits biofilm formation [18,19]. As an edible natural photosensitizer, Cur can also generate ROS under light irradiation, triggering lipid peroxidation, DNA strand breaks, and protein oxidation, thereby exerting broad-spectrum bactericidal effects [17,20]. Compared with conventional antibiotics, Cur-mediated PDT offers the advantages of low toxicity, high selectivity, and reduced risk of resistance [21]. In addition to its antimicrobial potential, Cur demonstrates significant promise in wound healing. It accelerates tissue repair by scavenging ROS, downregulating proinflammatory cytokines such as TNF-α and IL-6, and upregulating angiogenic mediators including VEGF. These effects promote neovascularization and collagen deposition, thereby facilitating rapid and efficient wound closure [22,23,24].

Cur possesses broad pharmacological activities, yet its clinical translation is hindered by poor solubility, low bioavailability, rapid degradation, and weak tissue penetration. It is unstable under neutral pH and rapidly metabolized into inactive forms, resulting in diminished therapeutic efficacy [25,26,27]. Moreover, its limited permeability across the stratum corneum restricts therapeutic action in deeper tissues [28]. To address these barriers, advanced delivery systems such as nanoliposomes, metal–organic frameworks (MOFs), exosomes, and hydrogels have been developed, employing encapsulation and stimuli-responsive release strategies to achieve localized and sustained drug delivery [22,29]. Among them, liposomes stand out for improving stability, bioavailability, and targeting capacity [30]. Their strong affinity for bacterial membranes enhances local retention and penetration [31,32], while controlled release prolongs antimicrobial efficacy [33]. Incorporation of bile salt edge activators, such as sodium deoxycholate, further increases membrane flexibility and permeability, facilitating bacterial membrane disruption [34,35]. Encapsulation of Cur into lipid-based nanocarriers thus represents a promising approach to overcome its intrinsic limitations and significantly enhance therapeutic outcomes.

In this work, we designed a curcumin-loaded liposomal photodynamic therapy system (Lipo-Cur) for the localized treatment of bacterial infectious wounds. The physicochemical properties, photodynamic ROS generation, antibacterial activity, wound-healing potential, and biosafety of Lipo-Cur were systematically evaluated.

## 2. Material and Methods

### 2.1. Chemicals and Reagents

Cur (purity ratio; 99.0%) was purchased from Rhawn Chemical Technology Co., Ltd. (Shanghai, China). Soybean phosphatidylcholine (SPC), sodium deoxycholate (SDC), and chloroform were purchased from Solarbio (Beijing, China). Other AR-grade chemicals were obtained from Aladdin Biochemical Technology Co., Ltd. (Shanghai, China) and Sangon Biotech Co. Ltd. (Guizhou, China).

### 2.2. Cells and Animals

Human keratinocytes (HaCaT) and murine fibroblasts (L929) were obtained from the American Type Culture Collection (ATCC; Manassas, VA, USA). *S. aureus* (ATCC 6538) and *E. coli* (ATCC 8739) were selected for antibacterial assays. Male KM mice (6–8 weeks old, 20–25 g) were purchased from Chengdu Dossy Experimental Animals Co., Ltd. (Chengdu, China).

### 2.3. Preparation of Lipo and Lipo-Cur

Lipo-Cur was synthesized using the thin-film dispersion and ultrasonication method. To optimize stability and encapsulation efficiency, SPC and SDC were dissolved in chloroform at a 5:1 ratio to prepare blank liposomes. Cur (0.5–10 mg) was then added and dissolved, and the mixture was subjected to rotary evaporation to form a uniform lipid film. The film was hydrated with PBS (pH 7.5) for 2 h and subsequently ultrasonicated in an ice-water bath at 400 W with 9 s on/3 s off cycles for a total of 10 min. The resulting homogeneous liposomes were stored at 4 °C until further use.

### 2.4. Material Characterization and Analysis

#### 2.4.1. Physicochemical Characterization

The physicochemical properties of Lipo-Cur were characterized using a series of complementary techniques. The morphology of Lipo and Lipo-Cur was examined by transmission electron microscopy (TEM), and their particle size distribution was measured by dynamic light scattering (DLS). Zeta potentials were determined to evaluate the surface charge and colloidal stability of the formulations.

To verify curcumin loading, the UV-Vis absorption spectra of Cur, Lipo, and Lipo-Cur were recorded from 200 to 800 nm, with particular attention to the characteristic absorption band of Cur and any peak shift after encapsulation. Fourier-transform infrared spectroscopy (FT-IR) was further performed to assess possible interactions between Cur and the liposomal matrix. Lyophilized samples of Cur, Lipo, and Lipo-Cur were mixed with potassium bromide (KBr) and pressed into pellets. Spectra were collected over the range of 4000–400 cm^−1^ at a resolution of 4 cm^−1^, and changes in the major functional-group signals were analyzed.

The dispersion behavior of the formulations was also visually assessed using the Tyndall effect. Briefly, Lipo and Lipo-Cur suspensions were placed in transparent glass vials and irradiated with a laser pointer in a dark room. The light path was photographed to provide a direct indication of nanoparticle formation and dispersion status.

#### 2.4.2. Encapsulation Efficiency and Release of Cur

Encapsulation efficiency (EE%) was determined by centrifugation and quantified using UV spectrophotometry based on a standard calibration curve. In vitro release profiles were obtained with the dialysis bag method (MWCO: 10 kDa) in PBS, and Cur concentrations were monitored by UV-Vis spectrophotometry. Transdermal absorption was evaluated by applying formulations to mouse dorsal skin followed by confocal laser scanning microscopy to assess epidermal penetration.

#### 2.4.3. Evaluation of Light-Triggered ROS Generation

ROS generation by Lipo-Cur under light irradiation (Lipo-Cur-BL^+^) was evaluated using electron paramagnetic resonance (EPR) spectroscopy. For further confirmation, a pH-based indirect assay was conducted. Briefly, free Cur and Lipo-Cur were added to 10% hydrogen peroxide (H_2_O_2_) solutions and divided into light-irradiated (BL^+^) and dark control (BL^−^) groups. The pH values were measured at 0, 2, 4, 6, 8, and 10 min after irradiation.

### 2.5. In Vitro Antibacterial Assays

The antibacterial activity of Lipo-Cur-BL^+^ was evaluated against drug-resistant *S. aureus* and *E. coli*. Bacterial suspensions (10^8^ CFU/mL) were incubated with different Cur-equivalent concentrations (0, 12.5, 25, 50, 100, and 200 μg/mL). Two conditions were applied: light irradiation (430 nm, 100 mW/cm^2^, 10 min) and dark controls. Bacterial growth was monitored by measuring the optical density at 600 nm (OD_600_) using a microplate reader at predetermined time points (0, 2, 4, 6, 12, 24, and 48 h), and growth curves were plotted. Colony-counting assays were performed to determine bactericidal activity. Treated suspensions were serially diluted with PBS (10–10^5^-fold) and plated on Luria–Bertani (LB) agar. Plates were incubated at 37 °C for 18–24 h, after which colony-forming units (CFUs) were enumerated to calculate bactericidal rates.

Bacterial membrane integrity was assessed using a SYTO-9/PI live/dead staining kit (Invitrogen, Carlsbad, CA, USA). SYTO-9 (green fluorescence) and propidium iodide (PI, red fluorescence) were used to label viable and nonviable cells, respectively. Images were acquired with a fluorescence microscope (Leica DMi8; Leica Microsystems, Wetzlar, Germany). For ultrastructural analysis, bacterial samples were fixed with 2.5% glutaraldehyde, dehydrated through a graded ethanol series, sputter-coated with gold, and imaged using scanning electron microscopy (SEM; SU8010, Hitachi High-Technologies Corporation, Tokyo, Japan).

### 2.6. In Vitro Cell Proliferation

#### 2.6.1. Cell Viability and Biocompatibility

929T and HaCaT were used to evaluate the biocompatibility of the Lipo-Cur nanosystem. Cell viability was assessed using the Cell Counting Kit-8 (CCK-8) assay. Cells were seeded in 96-well plates at a density of 5 × 10^3^ cells/well, cultured for 24 h, and treated with Lipo-Cur at Cur-equivalent concentrations of 2.5, 5, 10, 20, 40, and 80 μg/mL for 24 h. Subsequently, 10 μL of CCK-8 solution (Solarbio, China) was added to each well and incubated for 2 h, and absorbance was measured at 450 nm using a microplate reader. Cytotoxicity was further examined by live/dead staining with Calcein-AM and propidium iodide (PI). Fluorescence images were acquired using a confocal laser scanning microscope (Leica TCS SP8; Leica Microsystems, Wetzlar, Germany), where viable and nonviable cells were visualized by green and red fluorescence, respectively.

#### 2.6.2. Cell Proliferation and Migration

Cell proliferation was evaluated using the 5-ethynyl-2′-deoxyuridine (EdU) incorporation assay. Cells were treated with Lipo-Cur, and incubated with Click-iT^TM^ EdU reagent (Beyotime Biotechnology, Shanghai, China). Images were obtained, and EdU-positive cells were quantified. Cell migration was assessed using a wound-healing assay. Images of the wound areas were captured at 0 and 48 h, and wound closure was quantified using ImageJ software (version 1.46; National Institutes of Health, Bethesda, MD, USA). Colony formation assay was conducted to assess long-term proliferative capacity. Cells were seeded in 6-well plates at low densities (2000 and 4000 cells/well), treated with Lipo-Cur, and counted to determine colony numbers and sizes.

### 2.7. In Vivo Antibacterial Performance and Biosafety Evaluation

#### 2.7.1. Antibacterial Efficacy and Wound Healing

An *S. aureus*-infected wound model was established in Kunming (KM) mice. After infection stabilization, mice were randomly divided into six groups, with 6 mice in each group and a total of 36 mice used in this experiment. All animal procedures complied with the Regulations for the Administration of Affairs Concerning Experimental Animals (Ministry of Health, China) and were approved by the Animal Care and Use Committee of Kunming Medical University. Mice were anesthetized with isoaminobarbital (3%, *w*/*v*) intraperitoneal injection, and a 1.0 cm full-thickness circular wound was created dorsally. Wounds were inoculated with 40 μL S^−1^. aureus suspension (1 × 10^8^ CFU/mL). After infection stabilization, mice were divided into Control, Cur, and Lipo-Cur groups, with or without light irradiation (430 nm, 100 mW/cm^2^, 10 min). Wounds were photographed on days 0, 3, 6, 9, and 12. Wound areas were quantified using ImageJ, and healing curves were generated. On day 5, wound exudates were collected, plated on LB agar, and CFUs were counted. On day 12, mice were sacrificed, and wound-edge tissues were harvested for histological analysis. H&E staining was performed to examine inflammatory infiltration, epidermal regeneration, and granulation tissue formation. Masson’s trichrome staining was used to assess collagen deposition and organization.

#### 2.7.2. Systemic Biosafety

Major organs (heart, liver, spleen, lung, kidney) were collected post-treatment, paraffin-embedded, sectioned, and stained with H&E to evaluate pathological changes. Hemocompatibility was tested by hemolysis assay. Whole blood (1–2 mL) was collected from the retro-orbital plexus, centrifuged (2000 rpm, 15 min), washed with saline, and resuspended to 2% (*v*/*v*) RBCs. Diluted Lipo-Cur (0–80 μg/mL) was incubated with 1 mL RBC suspension at 37 °C for 3 h. Supernatants were collected after centrifugation, and absorbance at 540 nm was measured (Jingke 722N, Shanghai, China). Saline and distilled water served as negative and positive controls. Hemolysis was calculated as:

Hemolysis (%) = (AS − AN)/(AP − AN) × 100%

where AS, AN, AP are absorbances of sample, saline, and water controls, respectively. A hemolysis rate below 5% was considered acceptable. Body weights were recorded every two days throughout the treatment as an indicator of systemic toxicity and physiological stress.

### 2.8. Statistical Analysis

Data were analyzed using GraphPad Prism 10.3.1, OriginPro 2018, and ImageJ software. Results are expressed as mean ± standard deviation (SD). Statistical comparisons between groups were performed using one-way analysis of variance (ANOVA) and Student’s t-test. Differences were considered statistically significant at *p* < 0.05.

## 3. Results

### 3.1. Characterizations of Lipo and Lipo-Cur

**Figure 1:**
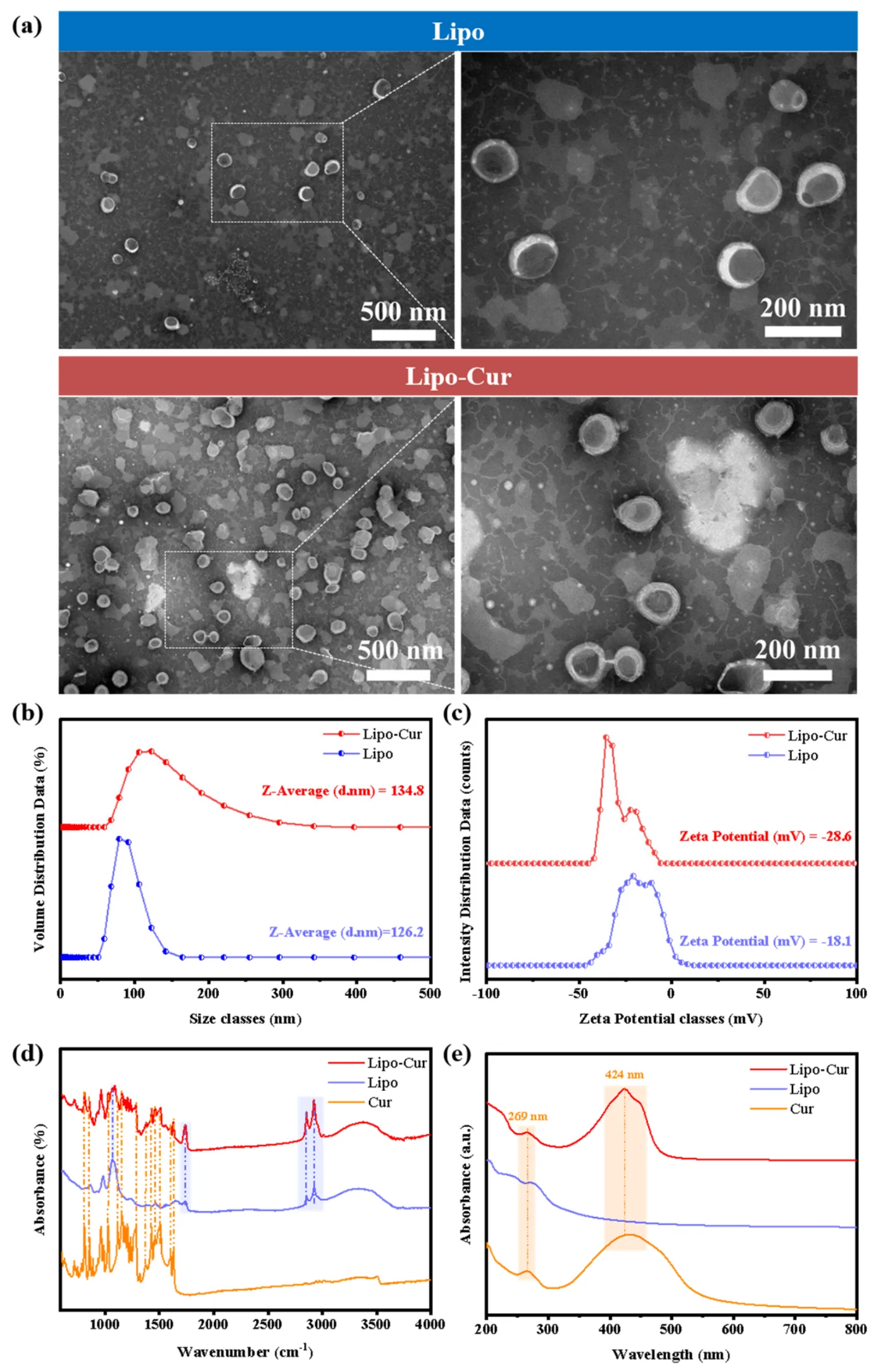
Morphology and chemical constitution of Lipo and Lipo-Cur. (**a**) TEM images of Lipo and Lipo-Cur; (**b**) hydrodynamic diameters of Lipo and Lipo-Cur; (**c**) zeta potentials of Lipo and Lipo-Cur; (**d**) FTIR spectra of Cur, Lipo, and Lipo-Cur; (**e**) UV-Vis absorption spectra of Cur, Lipo, and Lipo-Cur.

Lipo-Cur was fabricated using the thin-film dispersion-ultrasonication method. TEM analysis revealed that both Lipo and Lipo-Cur exhibited uniform, nearly spherical vesicles with clear boundaries and typical hollow structures (Figure 1a). No discernible morphological differences were observed between the two formulations, indicating that Cur encapsulation did not compromise the structural integrity or stability of the liposomes.

DLS measurements showed mean particle sizes of 126.2 nm for Lipo and 134.8 nm for Lipo-Cur (Figure 1b), suggesting a slight increase in size upon drug incorporation. Zeta potential values were −28.6 mV and −18.1 mV, respectively (Figure 1c), demonstrating that Cur loading altered the surface charge distribution. Fourier-transform infrared spectroscopy (FTIR) spectra provided further evidence of molecular interactions between Cur and the lipid bilayer (Figure 1d). Free Cur displayed characteristic absorption bands in the 1000–1700 cm^−1^ region, including an aromatic C=O stretching vibration at 1603 cm^−1^. Additional peaks at 1628, 1429, 1233, and 1115 cm^−1^ corresponded to aromatic C=C stretching, CH_2_-enol bending, C-CH, and in-plane aromatic C-CH vibrations, consistent with previous reports [36,37]. For blank liposomes, distinctive bands were detected at 1730 cm^−1^ (ester CO stretching), 1240–1100 cm^−1^ (P=O and C-O stretching), and 2850–2920 cm^−1^ (C-H stretching), characteristic of fatty acid chains [38,39]. In Lipo-Cur, the CO peak of Cur was markedly weakened and slightly red-shifted, whereas the lipid-associated C-H peaks remained unchanged. This spectral change indicates successful drug encapsulation within the bilayer, stabilized by hydrogen bonding or hydrophobic interactions without disrupting the liposomal structure. The absence of additional peaks further confirmed that the complex was formed predominantly through noncovalent interactions. UV-Vis spectra supported these findings. Cur exhibited two characteristic absorption maxima at 269 nm and 424 nm, assigned to π→π transitions of the aromatic system and n→π transitions of the conjugated diketone moiety, respectively [40,41]. In Lipo-Cur, the absorption at 424 nm shifted slightly to longer wavelengths and decreased in intensity, while short-wavelength absorption was enhanced and broadened. These changes reflect conformational adjustments of Cur and its interactions within the liposomal hydrophobic microenvironment (Figure 1e). Collectively, the results confirm efficient Cur encapsulation, structural stabilization, and improved dispersibility afforded by the liposomal carrier.

**Figure 2:**
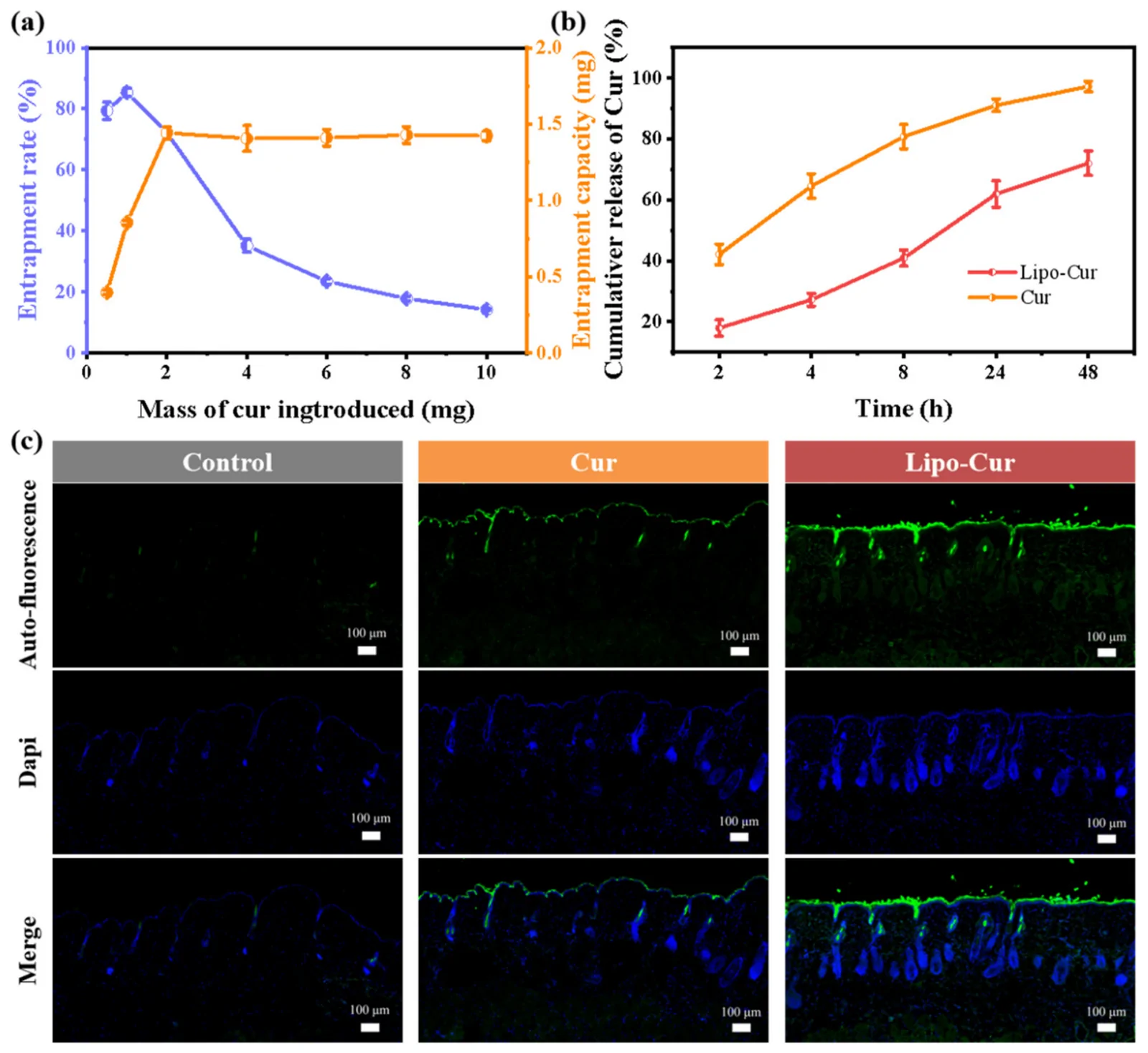
Characterization of the drug-loading and release properties of Lipo-Cur: (**a**) encapsulation efficiency of Cur in Lipo-Cur; (**b**) release profile of Cur from Lipo-Cur; (**c**) transdermal absorption of Cur and Lipo-Cur.

The Tyndall effect demonstrated that both Lipo and Lipo-Cur formed stable colloidal dispersions, whereas no visible light path was observed in PBS, confirming their excellent dispersion stability (Figure S1). The drug loading and release behavior of Lipo-Cur was subsequently investigated, with the calibration curve presented in Figure S2. Results indicated that when the feeding amount of Cur exceeded 1.5 mg, the actual encapsulated content reached a plateau, suggesting a maximum loading capacity of approximately 1.5 mg within the liposomal hydrophobic domain (Figure 2a). Under these conditions, the highest encapsulation efficiency achieved was 85.4 ± 1.38%. In vitro release studies showed that Lipo-Cur exhibited a sustained-release profile, with a cumulative release of 72.84 ± 1.89% at 48 h, which was markedly superior to free Cur suspension, indicating its potential to prolong drug action (Figure 2b). Confocal microscopy was further employed to assess the transdermal penetration of Cur. As a fluorescent molecule, Cur emits fluorescence under 404 nm excitation; however, untreated mouse skin also generates intrinsic autofluorescence due to endogenous fluorophores such as elastin and collagen [42]. Compared with the control, fluorescence intensity increased by 310% in the Cur suspension group and 650% in the Lipo-Cur, with the strongest signal observed in the latter, demonstrating that liposomal encapsulation significantly enhanced cutaneous penetration and accumulation (Figure 2c). Collectively, these findings confirm that Lipo-Cur possesses favorable transdermal penetration and sustained-release characteristics, validating its potential as a delivery platform for infectious wound therapy.

### 3.2. Photodynamic ROS Generation of Lipo-Cur

**Figure 3:**
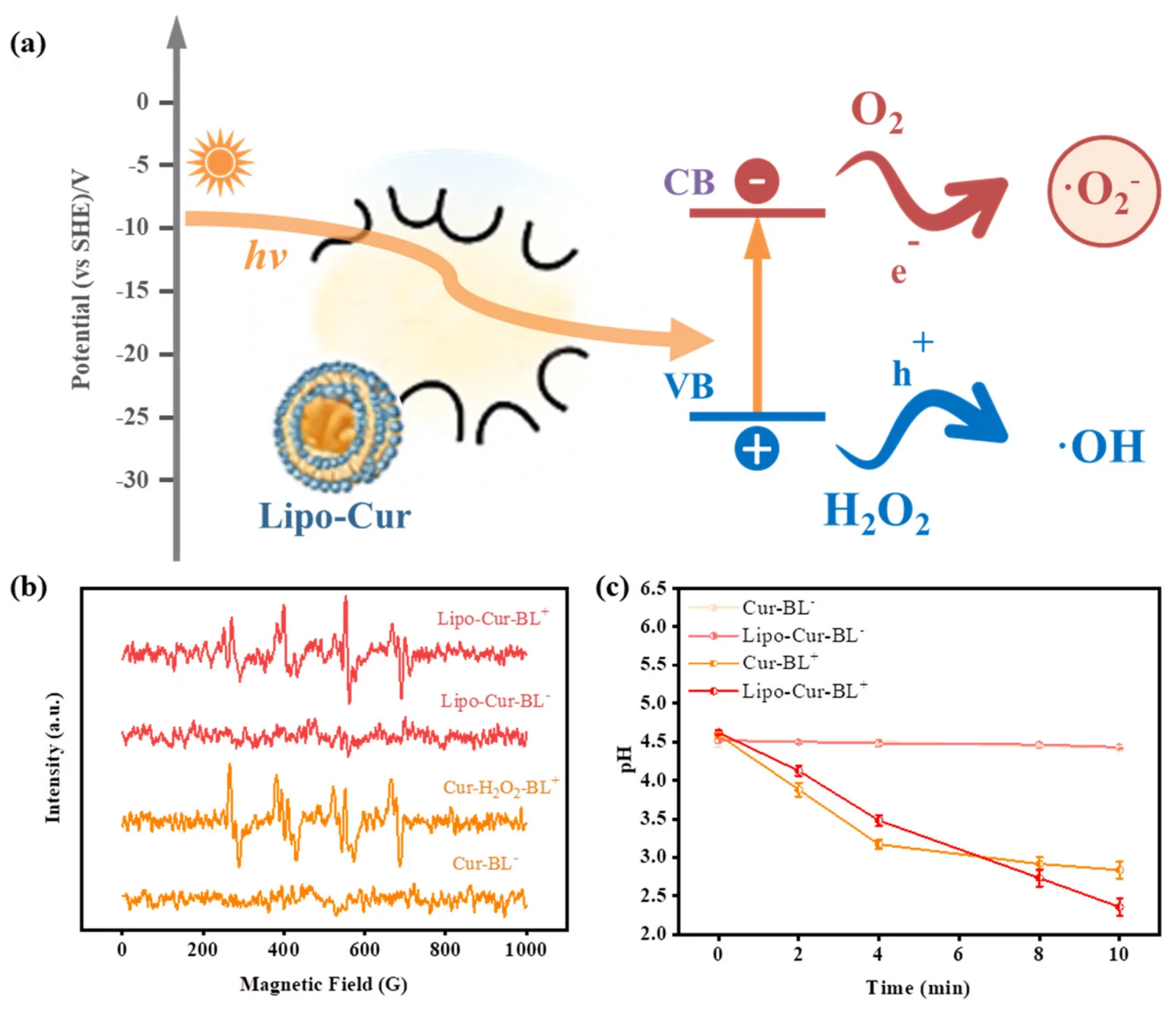
Characterization of the photodynamic reactive oxygen species (ROS) generation performance of Lipo-Cur. (**a**) Schematic illustration of the proposed photodynamic mechanism of Lipo-Cur, in which curcumin acts as the photosensitizer and the liposomal matrix improves its dispersibility and photostability under blue-light irradiation; (**b**) electron spin resonance (ESR) spectra of Cur and Lipo-Cur under BL^+^ and BL^−^ conditions; (**c**) temporal changes in pH for Cur and Lipo-Cur measured under BL^+^ and BL^−^ conditions.

Figure 3a illustrates the proposed photodynamic mechanism of Lipo-Cur under 430 nm blue-light irradiation. In this system, curcumin acts as the photoactive photosensitizer, while the liposomal matrix mainly serves as a carrier to improve its dispersibility, stability, and local availability. Upon light irradiation, encapsulated curcumin is excited and undergoes photoinduced energy- or electron-transfer reactions with surrounding oxygen-containing species, leading to the generation of reactive oxygen species (ROS), including superoxide radicals (O_2_•^−^) and hydroxyl radicals (·OH). The photosensitizing activity of Cur and Lipo-Cur was further examined by EPR spectroscopy (Figure 3b). In the absence of light, both samples showed signals close to baseline, indicating negligible background ROS. Under BL^+^, typical multiline splitting signals emerged for both Cur and Lipo-Cur, consistent with the formation of spin-trapped radicals like ·OH and O_2_^−^. Signal intensities were significantly stronger than those in the blank controls. Notably, the EPR signal of Lipo-Cur-BL^+^ was comparable to or slightly higher than that of free Cur, suggesting that liposomal encapsulation enhances curcumin’s dispersibility and reaction efficiency, thereby improving ROS generation.

To evaluate the light-triggered reactivity further, we monitored pH changes in 10% H_2_O_2_ solution under BL^+^ and BL^−^ (Figure 3c). Without light, the pH of Cur-BL^−^ and Lipo-Cur-BL^−^ remained nearly unchanged (around 4.5) over 10 min, indicating minimal ROS activity. In contrast, under light exposure, both samples showed a clear pH drop over time, confirming light-induced ROS production.

Interestingly, Cur-BL^+^ showed a sharp decline within the first 4 min, then plateaued, suggesting that Cur degrades rapidly when irradiated, but loses reactivity due to poor stability. Lipo-Cur-BL^+^, on the other hand, showed a more gradual yet sustained drop, with a greater decrease after 4 min compared to Cur. This indicates that the liposomal formulation slows initial degradation, improves photostability, and enables more continuous ROS release under light. These results highlight the potential of Lipo-Cur as a more stable and effective photodynamic agent for antimicrobial applications.

### 3.3. In Vitro Antibacterial Activity

**Figure 4:**
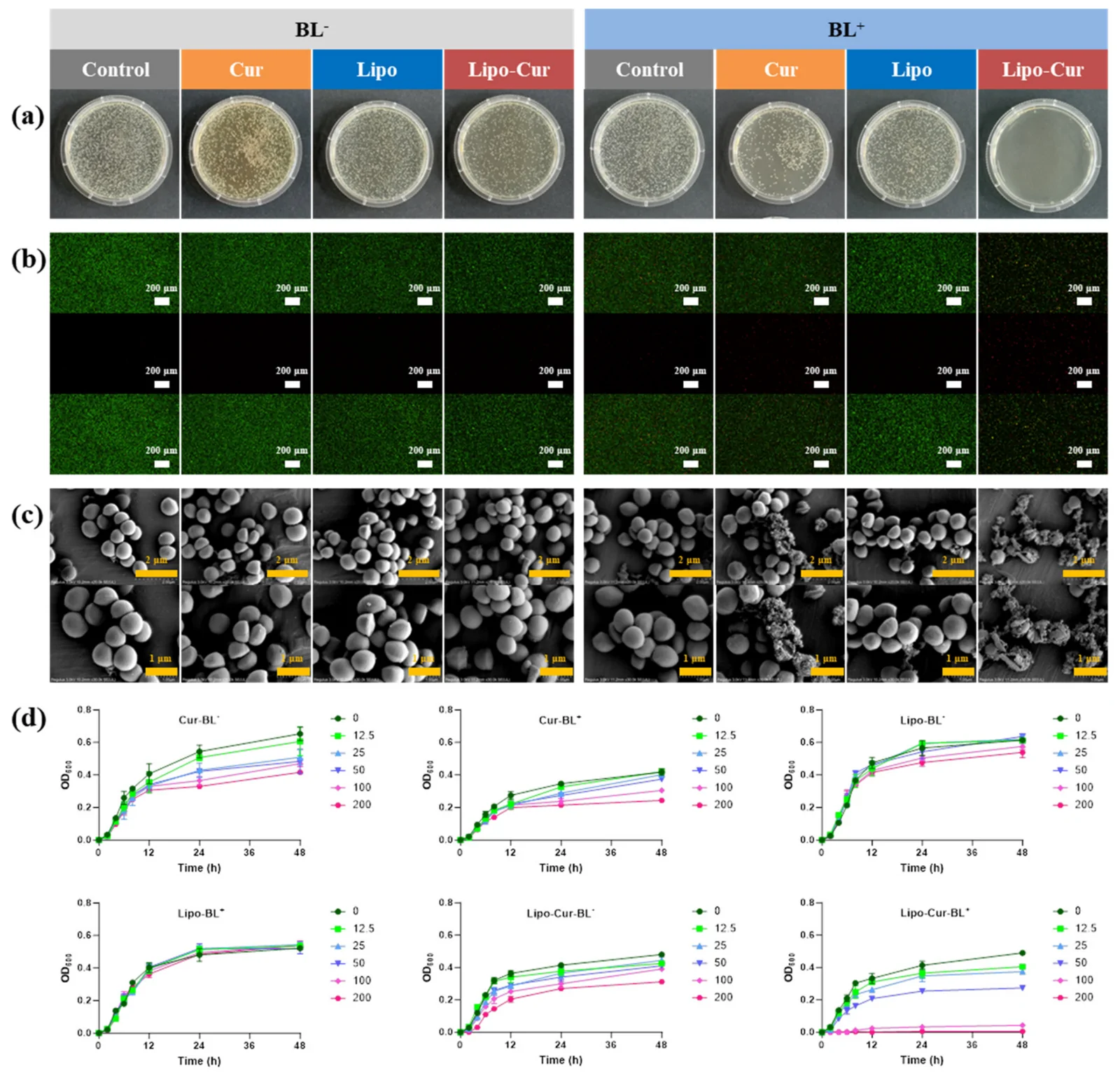
In vitro antibacterial activity of Lipo-Cur against *S. aureus*. (**a**) Representative images of *S. aureus* colonies on agar plates after different treatments; (**b**) Fluorescence images of live/dead-stained *S. aureus* (DMAO/PI) under each treatment condition. Scale bar: 200 μm; (**c**) SEM images showing morphological changes in *S. aureus* following various treatments. Scale bars: 1 μm and 2 μm; (**d**) Growth curves of *S. aureus* exposed to different concentrations of Cur, Lipo, and Lipo-Cur.

The antibacterial activity of Lipo-Cur was assessed using *S. aureus* and *E. coli* as representative Gram-positive and Gram-negative strains. Minimum inhibitory concentrations (MICs) were determined via the standard broth microdilution method, and bacterial growth was monitored by measuring OD_600_ [43]. As shown in Figure 4 and Figure S3, we compared the antibacterial effects of Lipo-Cur and Cur under both BL^+^ and BL^−^ conditions. Under Lipo-Cur-BL^+^ showed notably enhanced antibacterial activity, with MIC values of 100 μg/mL for *S. aureus* and 125 μg/mL for *E. coli*. These values were significantly lower than those observed in the Lipo, Cur, and Control groups (Figure 4d and Figure S3d). Growth curve analysis confirmed a clear concentration-dependent inhibition under BL^+^; at concentrations ≥ 100 μg/mL, Lipo-Cur almost completely suppressed bacterial growth (OD_600_ ≈ 0), while other formulations showed minimal or no effect. In contrast, no substantial antibacterial differences were found among groups in the absence of light. These results suggest that blue light significantly enhances the antibacterial efficacy of Cur, and that Lipo-Cur in particular benefits from a photo-induced mechanism with stronger potency.

To further investigate the bactericidal effects and underlying mechanisms of Lipo-Cur, several assays were conducted. Colony counting revealed that Lipo-Cur-BL^+^ achieved 100% killing of *S. aureus* and 98.26% for *E. coli* at MIC levels (Figure 4a, Figures S3a and S4), indicating complete bacterial clearance. Live/dead fluorescence staining showed strong red fluorescence signals after Lipo-Cur-BL^+^ treatment, corresponding to cell death rates of 95.04 ± 2.30% for *S. aureus* and 97.01 ± 3.45% for *E. coli* (Figure 4b, Figures S3b and S5). SEM imaging further supported these findings: *S. aureus* cells appeared shrunken and ruptured, while *E. coli* exhibited severe membrane disruption and cytoplasmic leakage after Lipo-Cur-BL^+^ exposure (Figure 4c and Figure S3c). Overall, these results indicate that Lipo-Cur-BL^+^ can effectively damage bacterial membranes, leading to loss of integrity, intracellular leakage, and cell death. This process is likely mediated by ROS generated during photodynamic activation.

### 3.4. Cytocompatibility, Proliferation, and Migration of Cells

**Figure 5:**
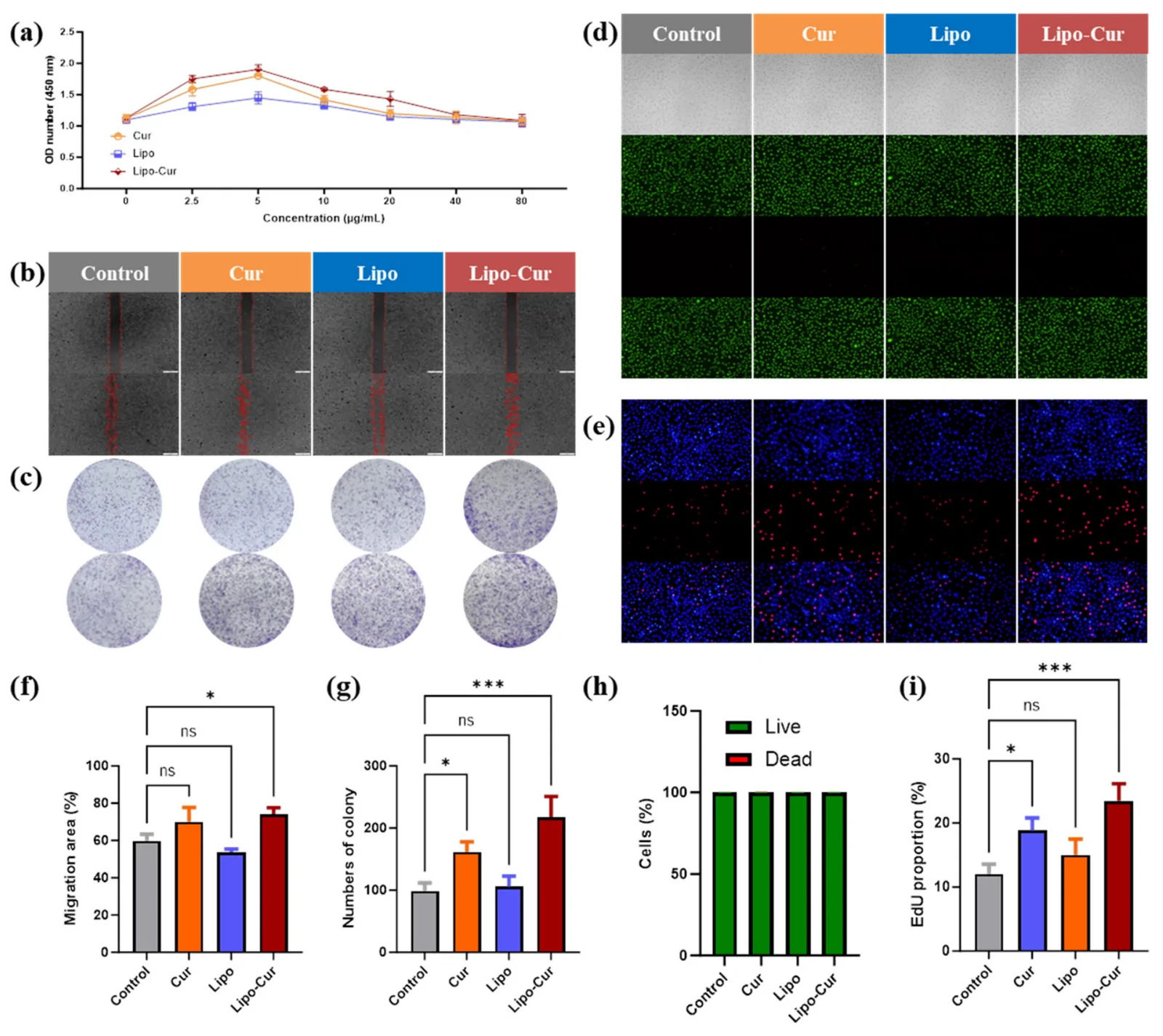
In vitro assessment of biocompatibility, cell migration, and proliferation of L929 cells. (**a**) Cell viability after 24 h incubation with different concentrations of Cur, Lipo, and Lipo-Cur, measured using the CCK-8 assay; (**b**) Cell migration and proliferation evaluated by scratch assay following treatment with the indicated formulations; (**c**) Colony formation assay to assess long-term proliferative capacity under various treatments; (**d**) CLSM images showing live/dead staining of L929 cells post-treatment, with live cells in green and dead cells in red; (**e**) EdU staining images indicating cell proliferation following different treatments. Scale bar: 50 μm; (**f**) Quantification of wound closure rates based on scratch assay images; (**g**) Statistical analysis of colony numbers across treatment groups; (**h**) Quantified live/dead cell ratios corresponding to panel; (**i**) Quantitative comparison of EdU-positive cells among the groups. Data are presented as mean ± SD. Statistical significance is indicated as follows: ns, not significant; * *p* < 0.05; *** *p* < 0.001.

The cytocompatibility, as well as the effects of Lipo-Cur on cell proliferation and migration, were assessed in vitro (Figure 5 and Figure S6). As shown in the CCK-8 assay (Figure 5a and Figure S6a), cells treated with Cur, Lipo, or Lipo-Cur showed higher OD_450_ values than the control, suggesting no obvious cytotoxicity. On the contrary, a mild stimulatory effect on cell proliferation or metabolism may be present. Among Cur, Lipo, and Lipo-Cur, Lipo-Cur led to the highest cell viability, significantly surpassing both free Cur and Lipo, indicating that liposomal encapsulation improves Cur uptake and bioavailability. Its sustained-release profile may also contribute to this enhanced proliferative effect while reducing potential toxicity. At 5 μg/mL, Lipo-Cur demonstrated good biocompatibility and clear pro-proliferative activity in both L929 and HaCaT cells, and this concentration was used in subsequent assays. Live/dead staining (Figure 5b and Figure S6b) confirmed the safety of the formulation-after 24 h of Lipo-Cur treatment, the proportion of PI-positive (dead) cells showed no significant difference from the control, consistent with the CCK-8 results.

Wound healing assays (Figure 5c and Figure S6c) showed that Lipo-Cur significantly accelerated cell migration, with better results than Cur or Lipo alone. These findings support its role in promoting both proliferation and motility. Colony formation assays (Figure 5d and Figure S6d) further demonstrated enhanced long-term proliferative capacity. While the control and Lipo groups formed moderate colonies and the Cur group showed slight improvement, the Lipo-Cur group exhibited the largest and most numerous colonies, reflecting stronger clonogenic potential. This pattern mirrored the migration results, suggesting that Lipo-Cur supports both short- and long-term cell proliferation.

EdU staining provided further evidence (Figure 5e and Figure S6e). Lipo-Cur treatment significantly increased EdU-positive cell ratios in both lines. In L929 cells, the EdU-positive rate rose to 23.43 ± 2.7% (vs. 12.03 ± 1.54% in control), and in HaCaT cells to 51.95 ± 3.4% (vs. 27.55 ± 2.49%), confirming enhanced DNA synthesis and proliferative activity. Across all assays-including CCK-8, live/dead staining, EdU incorporation, wound healing, and colony formation (Figure 5f–i and Figure S6f–i)-the Lipo-Cur group consistently outperformed both Cur and Lipo, with statistically significant differences. In summary, Lipo-Cur not only maintained excellent cytocompatibility but also significantly promoted cell growth and migration. These effects are likely due to improved cellular uptake and sustained release of Cur provided by the liposomal delivery system.

### 3.5. In Vivo Antibacterial Efficacy and Safety Evaluation

**Figure 6:**
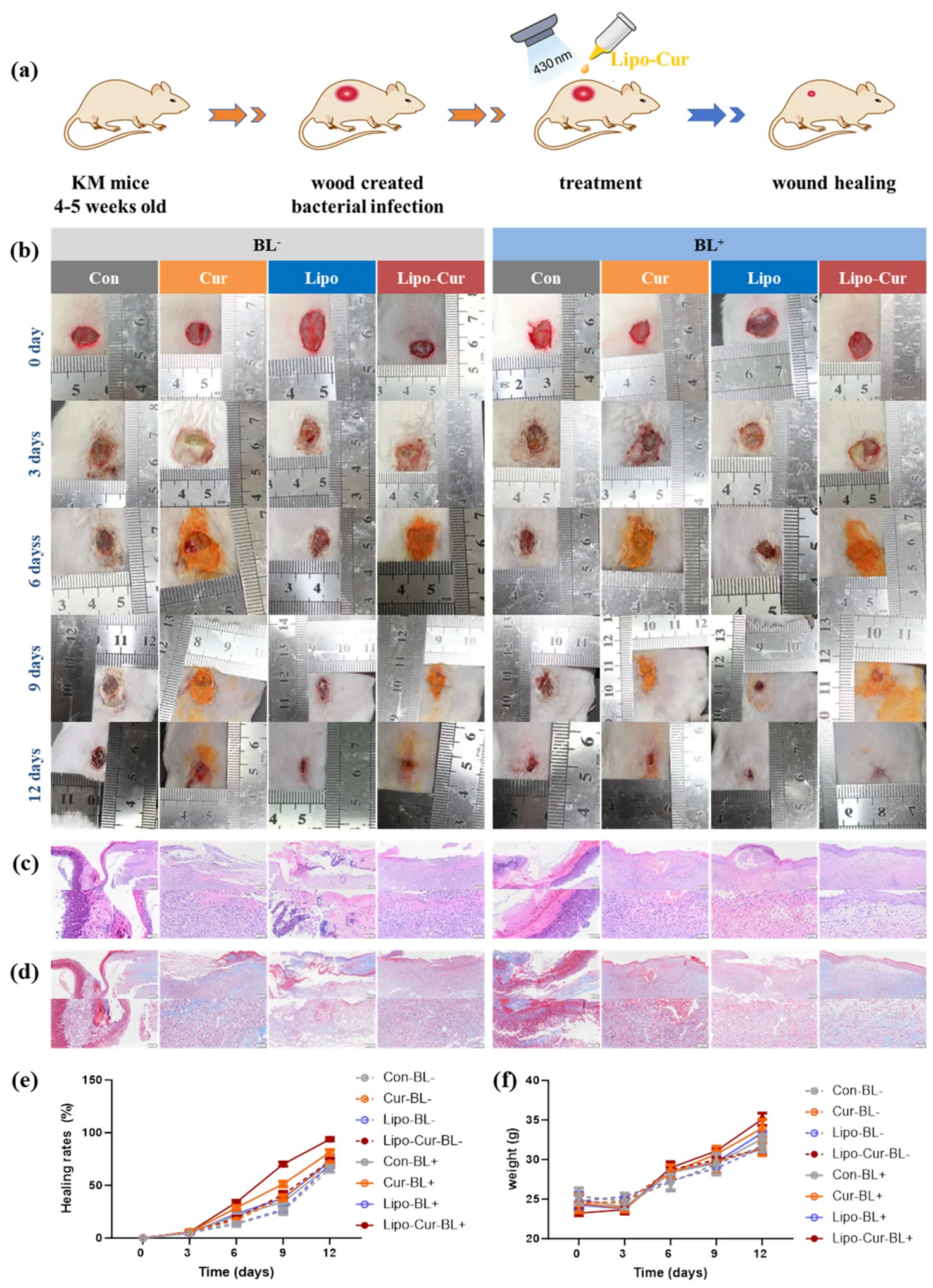
Therapeutic assessment of Lipo-Cur-BL^+^ in a mouse model of *S. aureus*-infected wounds. (**a**) illustration of the wound infection model induced by *S. aureus* and the corresponding treatment protocol; (**b**) representative images of infected wounds at days 0, 3, 6, 9, and 12 post-treatment; (**c**) H&E-stained tissue sections from each group on day 12, used for histological evaluation. Scale bars: 100 μm and 50 μm; (**d**) Masson’s trichrome staining of wound tissues collected on day 12, showing collagen deposition across groups. Scale bars: 100 μm and 50 μm; (**e**) quantification of wound closure rates across all treatment groups; (**f**) body weight changes in mice recorded during the treatment course.

The in vivo antibacterial performance and wound healing capacity of Lipo-Cur-BL^+^ were systematically assessed using a *S. aureus*-infected murine skin wound model. The Lipo-Cur-BL^+^ group demonstrated a clear therapeutic advantage throughout the healing process. Serial wound imaging and quantitative analysis revealed a noticeable wound contraction trend in the Lipo-Cur-BL^+^ group beginning on day 6 (Figure 6a), with a healing rate of 93.76 ± 1.56% by day 12, significantly higher than that of the control group (65.75 ± 3.18%, Figure 6b). Body weight monitoring (Figure 6f) indicated a mild initial weight loss post-infection, which recovered promptly after treatment. No signs of systemic toxicity or adverse effects were observed, suggesting good therapeutic efficacy and biocompatibility. Moreover, bacterial culture of wound exudates on day 5 (Figure S7) showed an antibacterial rate of 96.35 ± 2.51% in the Lipo-Cur-BL^+^ the most pronounced among all groups-demonstrating its potent in vivo antibacterial activity. Histological evaluations, including H&E and Masson’s trichrome staining, were further conducted to assess tissue regeneration. H&E staining (Figure 6c) showed extensive lymphocytic and neutrophilic infiltration and disrupted epidermal layers in the control group, whereas the Lipo-Cur-BL^+^ exhibited intact epithelial architecture and markedly reduced inflammation, indicating accelerated skin regeneration and effective inflammation control. Masson staining (Figure 6d) confirmed enhanced collagen deposition in the Lipo-Cur-BL^+^, with denser and more deeply stained collagen fibers, implying improved collagen synthesis associated with tissue remodeling.

Major organs (heart, liver, spleen, lung, kidney) were also examined by H&E staining (Figure S8a), revealing no signs of histopathological abnormalities or inflammatory infiltration, suggesting no systemic toxicity from the treatment. Hemocompatibility was further evaluated by hemolysis assays (Figure S8b–e), showing hemolysis rates below 5% for all concentrations of Lipo, Cur, and Lipo-Cur, with no visible erythrocyte rupture, confirming good blood compatibility of the materials. In summary, under BL^+^ irradiation, Lipo-Cur not only demonstrated strong antibacterial and wound healing-promoting effects on infected wounds but also showed high-quality tissue regeneration and excellent biosafety, highlighting its potential as a promising candidate for advanced wound care applications.

## 4. Discussion

Infected wounds, especially those caused by multidrug-resistant bacteria, remain difficult to manage clinically because of persistent biofilm formation, increasing antibiotic resistance, and delayed tissue repair. In this study, we developed a curcumin-loaded liposomal photodynamic nanosystem and demonstrated that it efficiently generated ROS under 430 nm blue-light irradiation. The system showed strong antibacterial activity against both Staphylococcus aureus and Escherichia coli, while also promoting the proliferation and migration of L929 fibroblasts and HaCaT keratinocytes. These combined effects contributed to faster healing of infected wounds with good biocompatibility. The findings indicate that Lipo-Cur acts not only as an antibacterial photosensitizer, but also as a bioactive platform that supports tissue repair, in line with the growing interest in antimicrobial photodynamic therapy for wound management [44].

Despite its well-documented antimicrobial and anti-inflammatory properties, the clinical use of curcumin has been limited by poor water solubility, low chemical stability, and insufficient bioavailability [29]. To address these limitations, we prepared an SPC/SDC (5:1)-based liposomal formulation using a thin-film dispersion and ultrasonication approach. The resulting nanoparticles exhibited an average diameter of approximately 130 nm and a zeta potential of −18.1 mV, indicating good colloidal stability. In addition, the formulation achieved a high encapsulation efficiency (85.4%) and sustained drug release over 48 h (72.8%). TEM, FTIR, and UV-Vis analyses confirmed that curcumin was successfully incorporated into the lipid bilayer, where a hydrophobic microenvironment helped stabilize the molecule and maintain controlled release behavior.

The antibacterial performance of Lipo-Cur-BL^+^ was primarily driven by ROS-mediated oxidative damage. EPR spectra verified the production of ·OH and O_2_^−^ species following blue-light exposure, while SEM imaging revealed membrane collapse, structural disruption, and cytoplasmic leakage in bacterial cells. These observations suggest that PDT exerts broad oxidative damage on bacterial membranes, proteins, and nucleic acids simultaneously, making the development of resistance less likely [45]. Notably, the MIC values obtained in this study (100–125 μg/mL) and the bactericidal efficiency (>95%) compared favorably with those reported for other curcumin-based PDT systems. Previous studies have shown that ROS can also disrupt biofilm matrices and improve antimicrobial penetration [46]. Although biofilm formation is an important clinical feature of chronic infected wounds, the present study focused on ROS generation and antibacterial activity against planktonic bacteria. Therefore, the current results should not be interpreted as direct evidence of antibiofilm efficacy. Future studies using established biofilm models are required to determine whether Lipo-Cur-BL^+^ can disrupt biofilm matrices, reduce biofilm-associated bacterial viability, or improve antimicrobial penetration within biofilm structures.

Beyond its antibacterial activity, Lipo-Cur also enhanced cell proliferation, migration, and colony formation. EdU incorporation and scratch assays showed increased DNA synthesis and migratory activity in fibroblasts and keratinocytes after treatment. Curcumin has been reported to facilitate wound repair through suppression of NF-κB-mediated inflammation, activation of the Nrf2/HO-1 antioxidant pathway, and upregulation of VEGF expression [47]. It is also possible that low levels of ROS generated during PDT triggered protective cellular responses that favored tissue regeneration. Consistent with the in vitro findings, in vivo experiments showed that the wound closure rate in the Lipo-Cur-BL^+^ group reached 93.76 ± 1.56% by day 12. Histological analyses further demonstrated reduced inflammatory infiltration and enhanced collagen deposition, without evidence of obvious systemic toxicity. Therefore, the enhanced antibacterial and wound-healing effects observed in the Lipo-Cur-BL^+^ group should be interpreted as resulting from the combined action of curcumin-mediated photodynamic therapy, rather than from curcumin alone. Although Lipo-Cur promoted fibroblast and keratinocyte proliferation and migration in vitro, the accelerated wound closure observed in the infected wound model may result from both direct pro-reparative effects and indirect benefits associated with bacterial elimination and reduced inflammation. Therefore, the in vivo effect of Lipo-Cur-BL^+^ should be interpreted as a combined antibacterial and wound-repair-supporting effect. Further studies are needed to distinguish its direct regenerative activity from infection-resolution-mediated healing.

Several limitations should nevertheless be considered. A limitation of this study is that in vitro cytocompatibility was mainly evaluated without blue-light irradiation. Because the antibacterial activity of Lipo-Cur-BL^+^ relies on light-triggered ROS generation, the response of mammalian cells under identical PDT conditions requires further investigation. Future studies should therefore assess PDT-related cytocompatibility in L929 and HaCaT cells under the same irradiation parameters. Moreover, the therapeutic efficacy and long-term biosafety of this platform still need to be evaluated in chronic wound models, including diabetic wounds, under repeated treatment conditions. The antibacterial efficacy of Lipo-Cur was evaluated only against standard laboratory strains, including *S. aureus* ATCC 6538 and *E. coli* ATCC 8739. Therefore, further studies using clinically isolated multidrug-resistant bacteria, such as methicillin-resistant *S. aureus*, are required to validate the broader applicability of this nanosystem for drug-resistant wound infections. Overall, the Lipo-Cur photodynamic nanosystem represents a promising local therapeutic strategy for drug-resistant infected wounds by combining effective antibacterial activity with wound-healing promotion.

## 5. Conclusions

The curcumin-loaded liposomal photodynamic system developed in this study improved the stability and local delivery efficiency of curcumin. Under blue light irradiation, the system continuously generated reactive oxygen species and showed strong antibacterial activity against both Staphylococcus aureus and Escherichia coli. In addition, it promoted the proliferation and migration of fibroblasts and keratinocytes, thereby accelerating infected wound healing. Animal experiments further demonstrated its ability to reduce bacterial burden, alleviate inflammation, and enhance tissue regeneration without obvious toxicity, suggesting promising potential for clinical application in infected wound treatment.

## Data Availability

The original contributions presented in this study are included in the article/Supplementary Material. Further inquiries can be directed to the corresponding authors.

## Supplementary Materials

The following supporting information can be downloaded at: https://www.mdpi.com/article/10.3390/pharmaceutics18091122/s1, Figure S1. Tyndall effect test of curcumin-loaded liposome dispersions. A red laser beam (650 nm) was sequentially passed through PBS, Cur, lipo, and Lipo-Cur. Figure S2. Standard calibration curve of curcumin. Absorbance at 424 nm was plotted against curcumin concentration, yielding a strong linear correlation with the regression equation y = 0.06046x + 0.03977, R^2^ = 0.9999. Figure S3. In vitro antibacterial activity characterization of Lipo-Cur against *E. coli*. (a) Representative photographs of *E. coli* colonies on solid agar plates after the indicated treatments. (b) Representative live/dead fluorescence images of *E. coli* stained with DMAO (live) and propidium iodide (PI, dead) following each treatment; scale bar = 200 μm. (c) SEM images of *E. coli* after the indicated treatments; scale bars = 1 μm and 2 μm. (d) Growth curves of *E. coli* exposed to different concentrations of Cur, Lipo, and Lipo-Cur. Figure S4. Quantitative analysis of bacterial survival by colony-forming assay. The percentages of colony formation for *S. aureus* (a) and *E. coli* (b) after the indicated treatments were quantified relative to the Control. Figure S5. Quantitative analysis of bacterial viability by live/dead staining. The proportions of live bacteria (green) and dead bacteria (red) were quantified for *S. aureus* (a) and *E. coli* (b) following the indicated treatments, and are presented as percentages of total bacteria. Figure S6. In vitro biocompatibility, migration, and proliferation assessments. (a) Cell viability of HaCaT cells after 24 h co-incubation with varying concentrations of Cur, Lipo, and Lipo-Cur, as measured by the CCK-8 assay. (b) Representative wound-healing (scratch) assay images used to evaluate HaCaT cell migration and proliferation following treatment with the different materials. (c) Colony formation assay assessing proliferative capacity of HaCaT cells after the indicated treatments. (d) Live/dead staining of HaCaT cells after treatments; live cells appear green and dead cells appear red. (e) Representative fluorescence images of EdU incorporation assessing HaCaT cell proliferation after treatment (scale bar = 50 μm). (f) Quantification of wound closure rate from the scratch assay. (g) Quantified colony counts for each treatment group. (h) Quantification of live/dead staining results. (i) Quantification of EdU-positive cells for each group. Figure S7. Representative photographs of bacterial colonies recovered on day 6 from periwound exudates collected from wounds treated with the different samples. Figure S8. In vivo safety evaluation. (a) H&E-stained sections of heart, liver, spleen, lung, and kidney harvested on day 12 (scale bar = 20 μm). (b–e) Hemolysis assays of erythrocytes following exposure to varying concentrations of Cur, Lipo, and Lipo-Cur; ultrapure water and PBS were used as the positive and negative controls, respectively.
