# Supplementary material for: A Curcumin-Loaded Liposomal Photodynamic Nanosystem for Effective Treatment of Bacterial Wound Infections
Source: Pharmaceutics. 2026 Sep 7;18(9):1122. doi: 10.3390/pharmaceutics18091122 (PMC13609951; doi:10.3390/pharmaceutics18091122)
Supplement: Supplementary file 1 [file pharmaceutics-18-01122-s001.zip › pharmaceutics-4380417-supplementary.pdf]

Support Information

A Curcumin-Loaded Liposomal Photodynamic Nanosystem for Effective Treatment of Bacterial Wound Infections

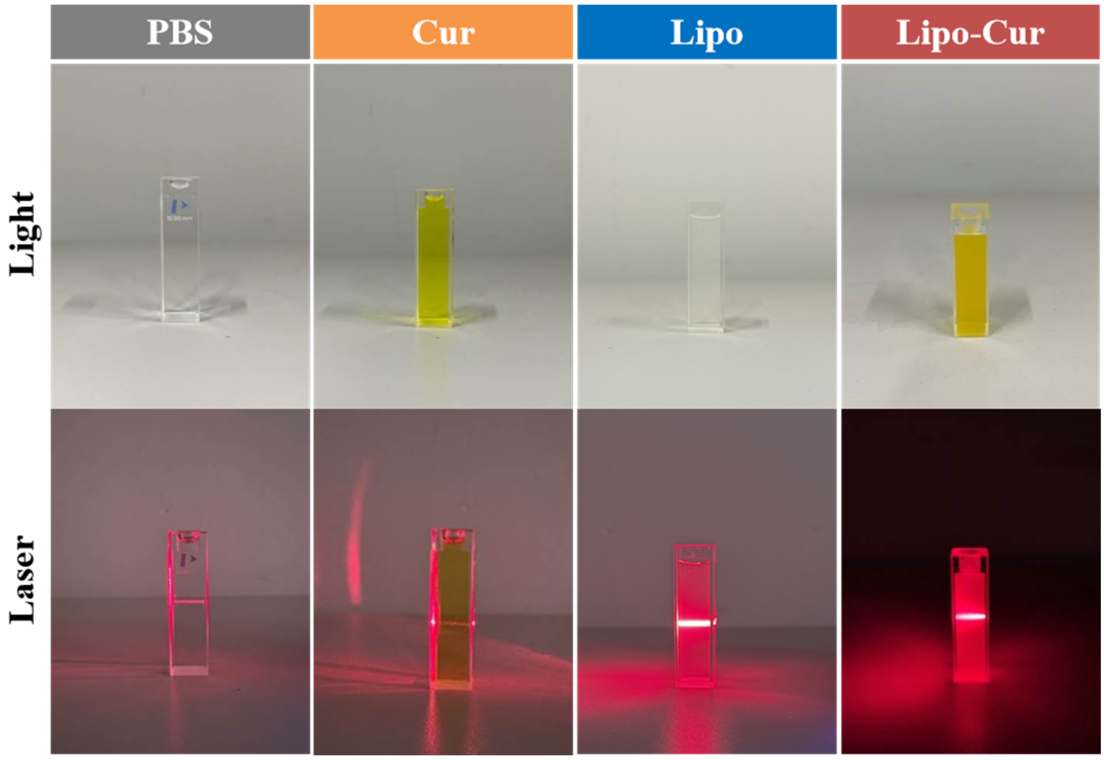

Figure S1. Tyndall effect test of curcumin-loaded liposome dispersions. A red laser beam (650 nm) was sequentially passed through PBS, Cur, lipo, and Lipo-Cur

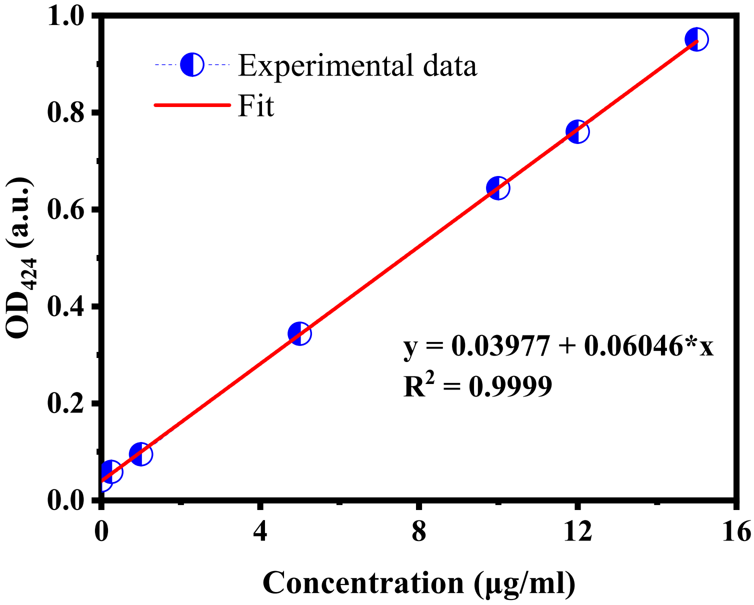

Figure S2. Standard calibration curve of curcumin. Absorbance at 424 nm was plotted against curcumin concentration, yielding a strong linear correlation with the regression equation y = 0.06046x + 0.03977, R^2 = 0.9999

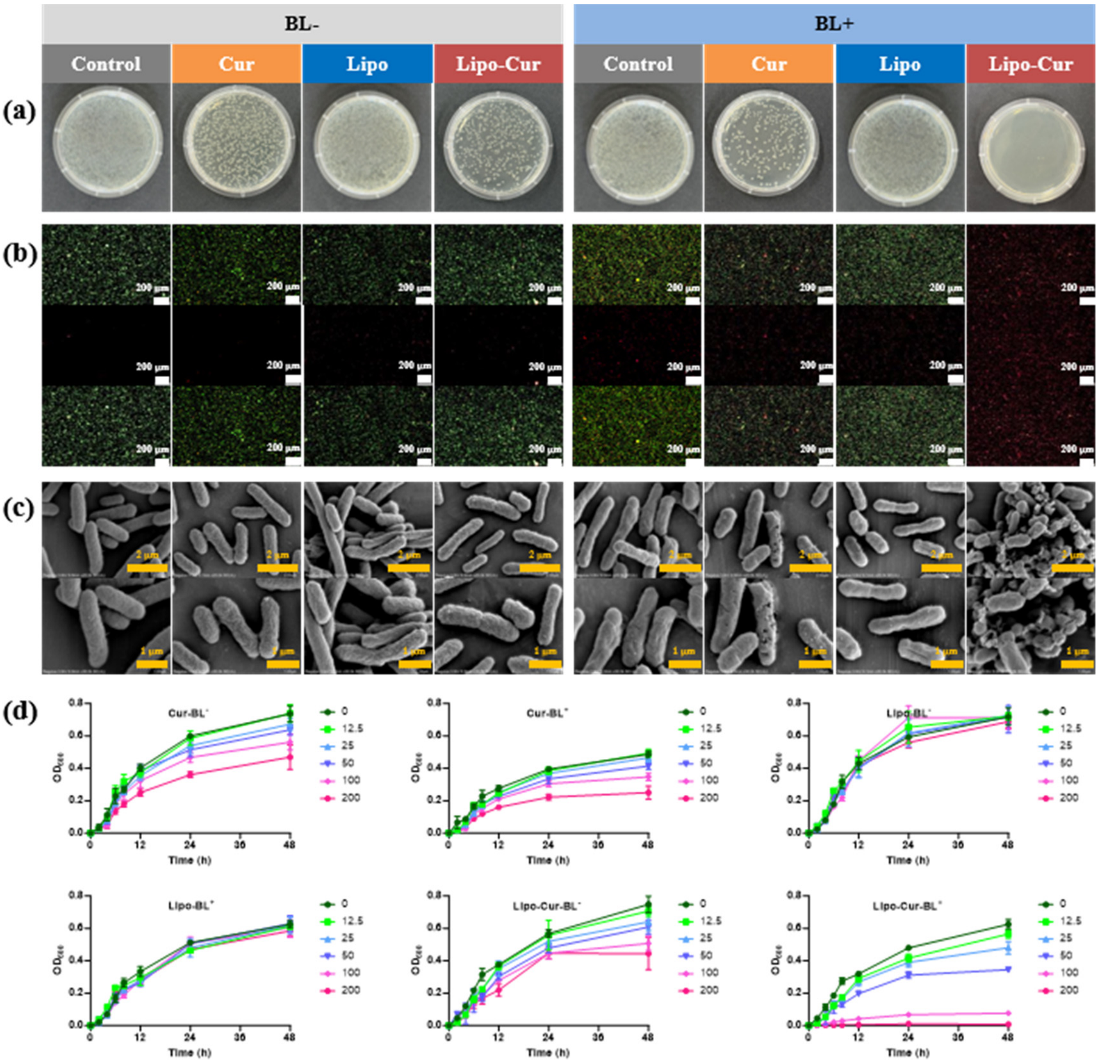

Figure S3. In vitro antibacterial activity characterization of Lipo-Cur against E.coli. (a) Representative photographs of E. coli colonies on solid agar plates after the indicated treatments. (b) Representative live/dead fluorescence images of E. coli stained with DMAO (live) and propidium iodide (PI, dead) following each treatment; scale bar = 200 μm. (c) SEM images of E. coli after the indicated treatments; scale bars = 1 μm and 2 μm. (d) Growth curves of E. coli exposed to different concentrations of Cur, Lipo, and Lipo-Cur

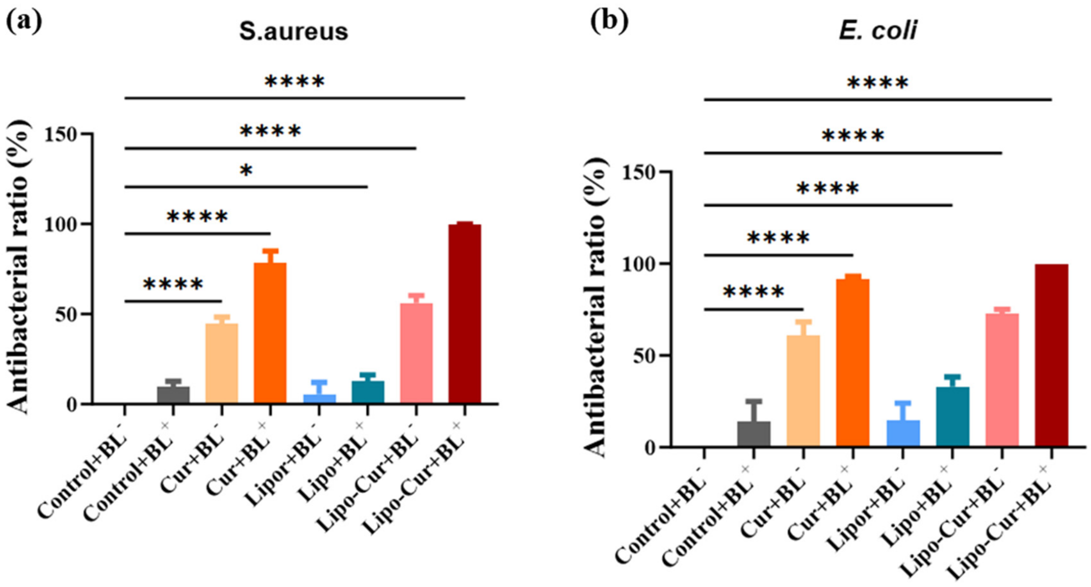

Figure S4. Quantitative analysis of bacterial survival by colony-forming assay. The percentages of colony formation for S. aureus (a) and E. coli (b) after the indicated treatments were quantified relative to the Control.

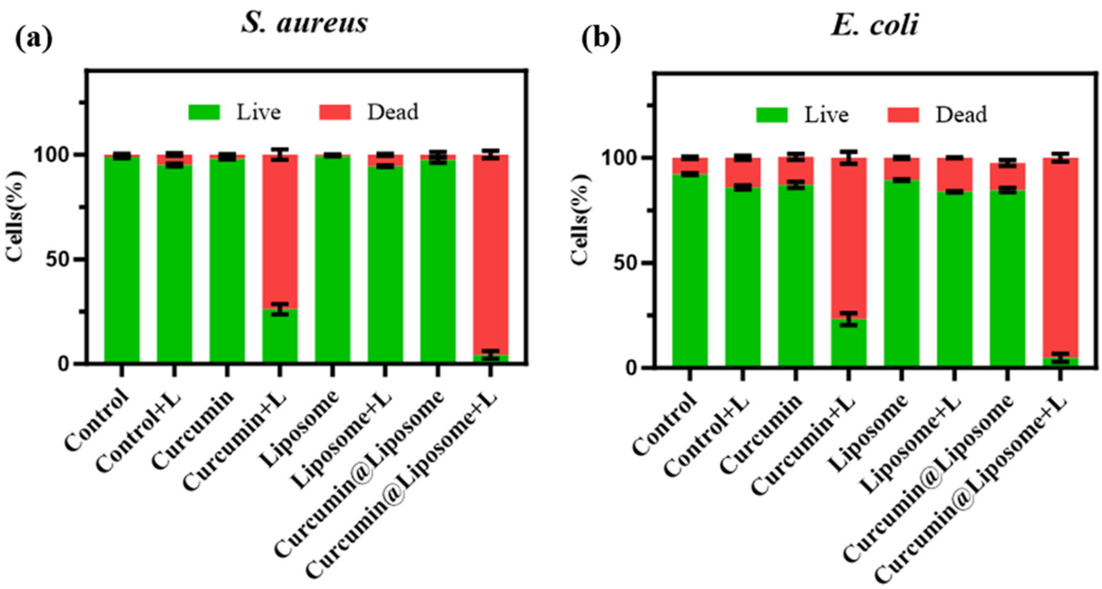

Figure S5. Quantitative analysis of bacterial viability by live/dead staining. The proportions of live bacteria (green) and dead bacteria (red) were quantified for S. aureus (a) and E. coli (b) following the indicated treatments, and are presented as percentages of total bacteria.

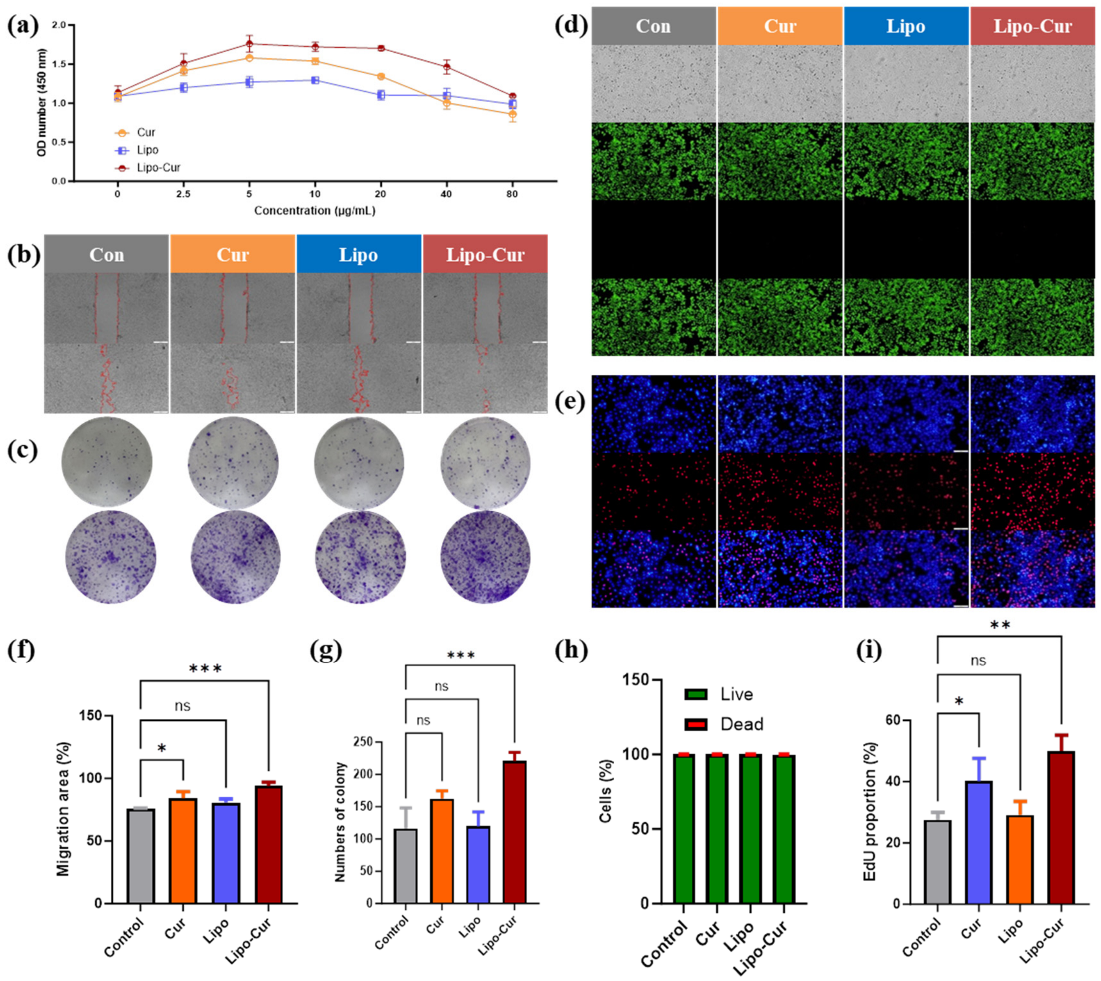

Figure S6. In vitro biocompatibility, migration, and proliferation assessments. (a) Cell viability of HaCaT cells after 24 h co-incubation with varying concentrations of Cur, Lipo, and Lipo-Cur, as measured by the CCK-8 assay. (b) Representative wound-healing (scratch) assay images used to evaluate HaCaT cell migration and proliferation following treatment with the different materials. (c) Colony formation assay assessing proliferative capacity of HaCaT cells after the indicated treatments. (d) Live/dead staining of HaCaT cells after treatments; live cells appear green and dead cells appear red. (e) Representative fluorescence images of EdU incorporation assessing HaCaT cell proliferation after treatment (scale bar = 50 μm). (f) Quantification of wound closure rate from the scratch assay. (g) Quantified colony counts for each treatment group. (h) Quantification of live/dead staining results. (i) Quantification of EdU-positive cells for each group

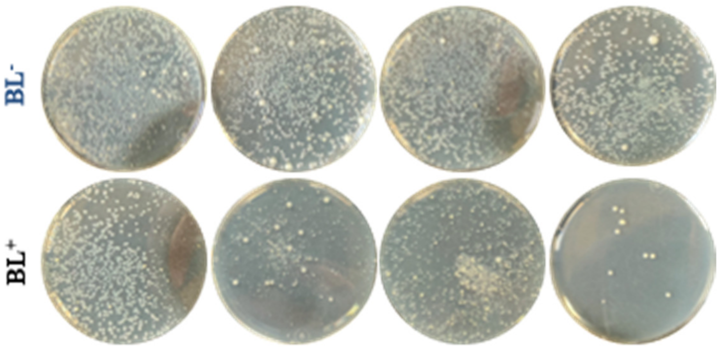

Figure S7. Representative photographs of bacterial colonies recovered on day 6 from periwound exudates collected from wounds treated with the different samples

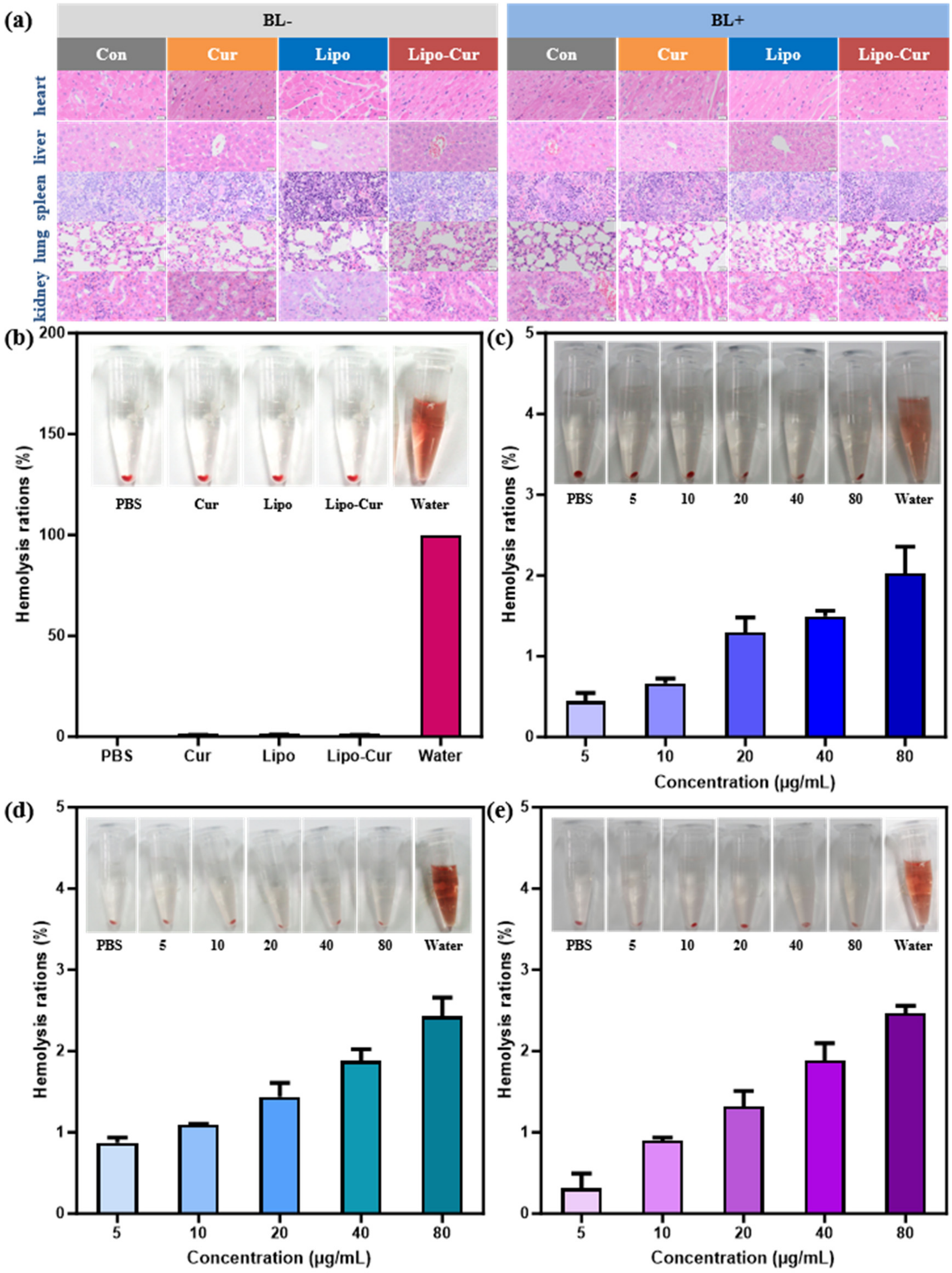

Figure S8. In vivo safety evaluation. (a) H&E-stained sections of heart, liver, spleen, lung, and kidney harvested on day 12 (scale bar = 20 μm). (b–e) Hemolysis assays of erythrocytes following exposure to varying concentrations of Cur, Lipo, and Lipo-Cur; ultrapure water and PBS were used as the positive and negative controls, respectively
